# ﻿Two new species of *Metacampanella* (Agaricales, Marasmiaceae) from China and Mongolia

**DOI:** 10.3897/mycokeys.108.131983

**Published:** 2024-09-10

**Authors:** Wei-nan Hou, Burenbaatar Ganbaatar, Tolgor Bau

**Affiliations:** 1 Key Laboratory of Edible Fungal Resources and Utilization (North), Ministry of Agriculture and Rural Affairs, Jilin Agricultural University, Changchun 130118, China Jilin Agricultural University Changchun China; 2 Laboratory of Plant Taxonomy and Phylogenetic, Botanic Garden and Research Institute, Mongolian Academy of Sciences, Ulaanbaatar 13330, Mongolia Botanic Garden and Research Institute, Mongolian Academy of Sciences Ulaanbaatar Mongolia; 3 School of Animal Science & Biotechnology, Mongolian University of Life Sciences, Ulaanbaatar 17024, Mongolia Mongolian University of Life Sciences Ulaanbaatar Mongolia

**Keywords:** *Metacampanella*, new combination, new species, phylogeny, taxonomy

## Abstract

*Metacampanella* is an important genus in the Marasmiaceae family. We collected specimens during our investigations in China and Mongolia. Through morphological and molecular phylogenetic analyses, we identified two new species of this genus: *Metacampanellasubtricolor* and *Metacampanellacoprophila*. In addition, we identified *Metacampanellatricolor* as a novel combination. Molecular systematic studies support these results. Illustrated descriptions, taxonomic discussions, and keys to the genus are provided.

## ﻿Introduction

*Metacampanella* is a genus in the Marasmiaceae family. Initially, species of this genus were placed in *Tetrapyrgos* and *Campanella* based on their morphological characteristics (Horak 1986; Kirk 2008). Hennings (1895) established the genus *Campanella* based on gelatinized trama, smooth, hyaline and inamyloid basidiospores and a pileipellis showing Rameales-structure or asterostromelloid layer (Singer 1975, 1986). Horak (1987) established the genus *Tetrapyrgos* based on small basidiomes with a pileus rarely greater than 10 mm diameter, a central to eccentric, black to bluish black stipe arising from a basal pad, a cutis-type pileipellis of loosely interwoven, diverticulate hyphae, cystidia with an often bulbous apex and diverticulate central axis, and hyaline, inamyloid, tetrahedral basidiospores. However, the species of the two genera could not be clearly distinguished solely based on the morphology, and the taxonomic statuses of some species remain unclear. These species were temporarily placed in one of the two genera. With the development of molecular technologies and availability of sequences, Honan et al. (2015) constructed a phylogenetic tree of these two genera and found that *Campanellasubdendrophora* was independent of *Tetrapyrgos* and *Campanella*, highlighting the issues with this classification (Komura et al. 2020). For a better distinction, Desjardin et al. (2017) re-described and provided the ITS and nrLSU sequences of important type species in these genera. Petersen and Hughes (2024) constructed a phylogenetic tree based on ITS and nrLSU fragments of *Tetrapyrgos*, *Campanella* and related taxa showing that *C.subdendrophora* and related taxa belonged to a third clade. *Metacampanella* was established to accommodate this clade. *Metacampanelladendrophora* (Singer) R.H. Petersen was identified as the type species of this genus. The species of this genus were characterized by basidiomata conchate, obcupulate to obsaucer-shaped, sessile, pseudostipitate or laterally stipitate, pileus surface matt; hymenophore usually of some radial rounded veins, usually developing few to numerous interveins, meandering or joining the major veins. Pileipellis were a thatch of slender hyphae and sometimes mixed with tetrapyrgoid pileocystidia, pleurocystidia fusiform, cheilocystidia missing, tetrapyrgoid or metuloid, with or without crystalline deposit. Fruiting on dead woody substrates, monocot, or uncommonly on dicot rotting wood.

At present, there are six species in this genus, *Metacampanellacaesia* (Romagn.) R.H. Petersen, *Metacampanellacostaricensis* R.H. Petersen, *Metacampanelladendrophora* (Singer) R.H. Petersen, *Metacampanellaolivaceonigra* (E. Horak) R.H. Petersen, *Metacampanellasinecystidia* R.H. Petersen and *Metacampanellasubdendrophora* (Redhead) R.H. Petersen.

Previously, our team had recorded and described the species of *Campanella* from the Changbai Mountain in Jilin Province, China (Bau and Liu 2010). Recently, we re-collected specimens from this series while conducting species diversity surveys in China and Mongolia. Through morphological observations and phylogenetic analyses, we identified two new species and a new combination of *Metacampanella*. This study aimed to conduct systematic macroscopic, microscopic, and molecular studies to provide a key to this genus.

## ﻿Material and methods

### ﻿Samplings and morphological analyses

Specimens for this study were collected from China and Mongolia. Specimens were deposited at the fungarium of Jilin Agricultural University (FJAU) as described in Cai et al. (2016) and Cui et al. (2018). The macroscopic description was based on fresh specimens in the field that were photographed, recorded and measured. The color description of basidiocarps was based on Kornerup and Wanscher (1978). The tissues of the specimens were treated with 5% KOH and 1% Congo red. Observations were made using a Carl Zeiss Primo Star optical microscope (Jena, Germany). The basidiospore measurements do not include the apiculus. They presented as length × width, ‘a–b × c–d’. ‘a–b’ and ‘c–d’ represents the minimum and maximum of 90% of the measured values. The main body (sterigmata or excrescences not included) of the basidia were presented as ‘e–f × g–h’. Cheilocystidia, caulocystidia, and pileipellis were measured (if present). A total of 40 mature spores were randomly selected from the specimens to measure the size and Q was the ratio of length divided by width. The description language and order refer to Petersen’s protocols when establishing the genus (Petersen and Hughes 2024).

### ﻿DNA extraction, PCR amplification, and sequencing

Genomic DNA was extracted by modified CTAB method (Doyle and Doyle 1987). DNA and PCR products were detected by 1% agarose gel electrophoresis (Larsson and Örstadius 2008). ITS1F(3’-CTTGGTCATTTAGAGGAAGTAA-5’) and ITS4 (5’-TCCTCCGCTTATTGATATGC-3’) was used as primers for ITS sequences amplification and sequencing (White et al. 1990). LR0R (5’ -ACCCGCTGAACTTAAGC-3’) and LR5 (5’-ATCCTGAGGGAAACTTC-3’) was used for nrLSU sequences amplification and sequencing primers (Vilgalys and Hester 1990). The polymerase chain reaction (PCR) procedures were carried out according to the protocol described by Mou and Bau (2021). Dideoxy sequencing was completed by Shenggong Bioengineering (Shanghai) Co., Ltd.

### ﻿Phylogenetic analyses

BioEdit was used to read new sequences (Alzohairy 2011), and DNAMAN (Lynnon Biosoft) was used to splice ITS and LSU sequences. New sequences were uploaded to the GenBank database National Center for Biotechnology Information (nih.gov). Sequences of related representative species in GenBank database and new sequences were selected to construct the phylogenetic tree (Table 1). Under the G-INI-I model, we used MAFFT 7.110 to align the sequence matrix https://mafft.cbrc.jp/alignment/server/. The Maximum Likelihood (ML) method used RAxML v8.2.4. The Bayesian (BI) phylogenetic tree was constructed using Phylosuite v1.2.2 (Zhang et al. 2020). Trees were displayed by FigTree v1.4.4. We referred to Petersen’s research results on species of Marasmiaceae (Petersen and Hughes 2024). 167 ITS sequences and 43 nrLSU sequences were used in the matrix of phylogenetic analysis. The length of ITS sequence matrix was 801, and the length of nrLSU sequence matrix was 1037. Maximum likelihood phylogenies were inferred using IQ-TREE under the edge-linked partition model for 10000 ultrafast bootstraps and the Shimodaira–Hasegawa-like approximate likelihood-ratio test. ModelFinder was used to select the best-fit model using AIC criterion. Best-fit model according to AIC: TPM2u+F+R3. Bayesian Inference(BI) phylogenetic tree was constructed by Phylosuite v1.2.2. ModelFinder (Kalyaanamoorthy et al. 2017). The best-fit partition model (Edge-linked) used BIC criterion. Best-fit model according to BIC: HKY+F+I+G4:ITS,HKY+F+I+G4.(Kalyaanamoorthy et al. 2017). In this study, *Marasmiellusrhizomorphogenus* Antonín, Ryoo & H.D. Shin was selected as the outgroup.

**Table 1.** GenBank accession number of sequences used in this study.

Taxon	Country	Collection	GenBank No.		Reference
			ITS	nLSU	
*Campanella* aff. *Buettneri*	China	TENN-F-050841ss3	OQ171234	OQ171234	Petersen and Hughes 2024
C.aff.pustulata	Australia	QMS0008	JX444165	—	—
*C.alba*	Unknown	ZMXR3	MT446108	—	—
*C.buettneri*	China	WEI17–513	MW527101	—	Wei et al. 2021
*C.buettneri*	San Tome and Principe	DED 8276 (SFSU) epitype	MF075136	MF075138	Desjardin et al. 2017
*C.buettneri*	China	SWFU 001873	MK809426	—	Guan and Zhao 2021
*C.buettneri*	Unknown	SFSU:AHH85	KT270852	—	Honan et al. 2015
*C.buettneri*	Unknown	SFSU:AHH14	KT270850	—	Honan et al. 2015
*C.buettneri*	Unknown	SFSU:AHH83	EF175518	—	Honan et al. 2015
*C.buettneri*	Unknown	SFSU:AHH72	EF175520	—	Honan et al. 2015
*C.buettneri*	Thailand	SFSU:AHH74	KT270851	—	Honan et al. 2015
*C.buettneri*	Thailand	SFSU:AHH42	EF175519	—	Honan et al. 2015
*C.buettneri*	China	TENN-F-051974	OQ171237	—	Petersen and Hughes 2024
*C.burkei*	São Tomé and Príncipe	SFSU:BAP 632	MF100970	—	Desjardin et al. 2017
*C.candida*	Cook Islands	PDD:102184	OQ282823	—	—
*C.keralensis*	India	AF 342	MW462889	—	—
*C.pustulata*	Australia	AQ793972	JX444168	—	—
*C.pustulata*	Australia	SMF2382	JX444164	—	—
*C.pustulata*	Australia	FBT2220	MW192636	—	
*C.simulans*	India	AF129	MW506836	—	—
*C.* sp.	Costa Rica	TENN-F-053828	OQ171240	OQ171240	Petersen and Hughes 2024
*C.* sp.	India	Strain JZ31	MG719288	—	—
*C.* sp.	India	JZ44	MG719301	—	—
*C.* sp.	USA	TENN-F-050996h1	OQ171235	—	Petersen and Hughes 2024
*C.* sp.	USA	TENN-F-050996h2	OQ171236	—	Petersen and Hughes 2024
*C.* sp.	Guyana	MCA1689	AY916670	AY916668	Aime and Phillips-Mora 2005
*C.* sp.	USA	MCA3234	MG717365	MG717352	Koch et al. 2018
*C.* sp.	New Zealand	PDD:96255	OQ282788	OQ282744	—
*C.* sp.	New Zealand	PDD:111968	OQ282827	OQ282774	—
*C.* sp.	New Zealand	PDD:112459	OQ282810	—	—
*C.* sp.	New Zealand	PDD:96318	OQ282789	OQ282789	—
*C.* sp.	Australia	HO:570075	OQ282798	OQ282753	—
*C.* sp.	Cook Islands	PDD:106889	OQ282807	OQ282760	—
*C.* sp.	Guyana	MCA2235	AY916676	AY916674	Aime and Phillips-Mora 2005
*C.* sp.	New Zealand	PDD:106900	OQ282809	—	—
*C.* sp.	New Zealand	PDD:106952	OQ282805	OQ282758	—
*C.* sp.	Polynesia	biocode 09–475	MZ997207	—	Osmundson et al. 2022
*C.tristis*	New Zealand	JAC9980	OQ282781	OQ282741	—
*C.tristis*	New Zealand	PDD:96329	OQ282790	OQ282746	—
*C.tristis*	New Zealand	PDD:104678	OQ282826	—	—
*C.tristis*	Australia	Clone Gs2A	FJ857922	—	Dearnaley and Bougoure 2010
*C.tristis*	Australia	Clone GS4A	FJ857925	—	Dearnaley and Bougoure 2010
*C.tristis*	Australia	Clone Gs3B	FJ857924	—	Dearnaley and Bougoure 2010
*Marasmielluscandidus*	USA	AHH157 (SFSU)	EF175513	—	Honan et al. 2015
*Ma.candidus*	Canada	UBC:F19683	HM240532	HM240532	—
*Ma.candidus*	Canada	UBC:F33072	MF908473	—	—
*Ma.candidus*	Canada	TENN-F-052592	OQ171238	OQ171238	Petersen and Hughes 2024
*Ma.candidus*	USA	TENN-F-068189	OQ171253	OQ171253	Petersen and Hughes 2024
*Ma.candidus*	USA	TENN-F-069193	OQ171256	OQ171256	Petersen and Hughes 2024
*Ma.candidus*	France	MICH175508	MN173348	—	—
*Ma.candidus*	Unknown	CBS252.39	MH856003	—	Vu et al. 2019
*Ma.candidus*	New Zealand	PDD:86918	OQ282779	—	—
*Ma.candidus*	New Zealand	PDD:112971	OQ282815	OQ282765	—
*Ma.candidus*	New Zealand	PDD:86983	OQ282780	OQ282740	—
*Ma.candidus*	India	KUBOT-KRMK-2020-72	MW485122	MW485123	Kantharaja and Krishnappa 2020
*Ma.candidus*	USA	BHI-F446d	MF161268	—	Haelewaters et al. 2018
*Ma.celebanticus*	SPAIN	TO HG2281 TYPE	NR_154152	—	Perez-de Gregorio et al. 2011
*Ma.rhizomorphogenus*	USA	RA705-27	MK234196	—	Alanbagi et al. 2019
*Metacampanellacaesia*	Mexico	Clone O7c81H	GQ924042	—	Herrera et al. 2010
*Me.caesia*	USA	Clone 8WE1cf02	GU910308	—	Herrera et al. 2010
*Me.caesia*	USA	Clone 8WE1cf06	GU910311	—	Herrera et al. 2010
*Me.caesia*	USA	Clone 8WE1cg01	GU910317	—	Herrera et al. 2010
*Me.caesia*	Kenya	Isolate F41	MW995635	—	Petersen and Hughes 2024
*Me.caesia*	USA	8WE3ch07	GU910438	—	Herrera et al. 2011
*Me.caesia*	Spain	T24	MH633918	—	Pereira,E. et al. 2019
*Me.caesia*	Kenya	CSB F175	KU680416	—	2024
*Me.caesia*	USA	Clone 8WE1cd05	GU910299	—	Herrera et al. 2010
*Me.caesia*	USA	Clone 8WE6cc07	GU910546	—	Herrera et al. 2010
*Me.caesia*	USA	Clone 8WE1cg12	GU910324	—	Herrera et al. 2010
*Me.caesia*	USA	Clone 8WE6ca04	GU910532	—	Herrera et al. 2010
*Me.caesia*	USA	Clone 8WE6cg02	GU910572	—	Herrera et al. 2010
*Me.caesia*	USA	Clone 8WE1cf11	GU910315	—	Herrera et al. 2010
*Me.caesia*	India	BROP8	KU168340	—	—
***Me.coprophila***	**Mongolia**	**FJAU69316**	**PP973101**	**PP973108**	**This study**
***Me.coprophila***	**Mongolia**	**FJAU69317**	**PP973102**	**PP973107**	**This study**
*Me.costaricensis*	Costa Rica	TFB9908ss13	OQ171249	OQ171249	Petersen and Hughes 2024
*Me.costaricensis*	Costa Rica	TENN-F-056536 Isotype	OQ171247	OQ171247	Petersen and Hughes 2024
*Me costaricensis*	Costa Rica	TENN-F-056536ss1	OQ171248	OQ171248	Petersen and Hughes 2024
*Me.dendrophora*	Argentina	TENN-F-055003ss4	OQ171243	OQ171243	Petersen and Hughes 2024
*Me.dendrophora*	Argentina	TENN-F-055002ss11	OQ171242	OQ171242	Petersen and Hughes 2024
*Me.olivaceonigra*	New Zealand	PDD:112550	OQ282811	OQ282761	—
*Me.olivaceonigra*	New Zealand	PDD:87486	OQ282784	—	—
*Me.olivaceonigra*	Australia	MEL2220682	EF175541	—	Honan et al. 2015
*Me.sinecystidia*	USA	C402M	KT203169	—	David et al. 2016
*Me.sinecystidia*	USA	AHH120 (SFSU)	EF175521	—	Honan et al. 2015
*Me.subdendrophora*	USA	iNAT-99991981	ON979424	—	—
*Me.subdendrophora*	USA	AHH79 (SFSU)	EF175523	—	Honan et al. 2015
*Me.subdendrophora*	Canada	ATCC 42449	AY445121	AY445115	Vinnere et al. 2005
*Me.subdendrophora*	Canada	ATCC 42449	NR_171206	NG_075153	Vinnere et al. 2005
*Me.subdendrophora*	USA	MushroomObserver.org/ 443698	MW433846	—	—
*Me.subdendrophora*	Mexico	TENN-F-055280	OQ171244	OQ171244	Petersen and Hughes 2024
*Me.subdendrophora*	USA	TENN-F-078187	OQ171257	—	Petersen and Hughes 2024
*Me.subdendrophora*	USA	DED7338 (SFSU)	EF175529	—	Honan et al. 2015
*Me.subdendrophora*	USA	AHH148 (SFSU)	EF175522	—	Honan et al. 2015
*Me.subdendrophora*	Canada	CCCM:UBC 5060-extype	OQ171258	OQ171258	Petersen and Hughes 2024
*Me.subdendrophora*	USA	TENN-F-059502	OQ171251	—	Petersen and Hughes 2024
***Me.subtricolor***	**China**	**FJAU69309**	**PP973106**	**PP973112**	**This study**
***Me.subtricolor***	**China**	**FJAU69310**	**PP973105**	**PP973111**	**This study**
***Me.tricolor***	**China**	**FJAU69313**	**PP973104**	**PP973110**	**This study**
***Me.tricolor***	**China**	**FJAU69314**	**PP973103**	**PP973109**	**This study**
*Me.tricolor*	Unknown	NN055704	JN943601	JN941149	Schoch et al. 2012
*Me.subdendrophora*	USA	UBC-F-33841b	OQ171259	—	Petersen and Hughes 2024
Root associated fungus	Australia	EP57	AY627833	—	Bougoure and Cairney 2005
T.aff.nigripes	Australia	MEL:2382866	KP012740	—	—
T.aff.nigripes	Australia	MEL:2382974	KP012833	—	—
*T.atrocyanea*	Brazil	INPA259598	KT287094	—	Komura et al. 2020
*T.atrocyanea*	Brazil	INPA259611	KT287095	—	Komura et al. 2020
*T.atrocyanea*	Brazil	INPA259597	KT287096	—	Komura et al. 2020
*T.atrocyanea*	India	KUBOT-KRMK-2020-80	MW555782	—	Kantharaja and Krishnappa 2022
*T.atrocyanea*	USA	TENN-F-055739	OQ171245	—	Petersen and Hughes 2024
*T.atrocyanea*	USA	FLAS-F-61224	MH211826	—	—
*T.atrocyanea*	Puerto Rico	TJB7935 (SFSU)	EF175544	—	Honan et al. 2015
*T.atrocyanea*	Bolivia	REHalling6376 (SFSU)	EF175533	—	Honan et al. 2015
*T.atrocyanea*	Costa Rica	REHalling8396 (SFSU)	EF175545	—	Honan et al. 2015
*T.atrocyanea*	Brazil	INPA259596	KT287093	—	Schoch et al. 2012
*T.atrocyanea epitype*	Madagascar	JES 216 (SFSU)	NR_169666	—	Desjardin et al. 2017
*T.brevicystidiata*	Brazil	DLK1065	KT287087	—	Komura et al. 2020
*T.brevicystidiata*	Brazil	INPA259604	KT287088	—	Komura et al. 2020
*T.cerebrata*	Brazil	INPA259594	KT287090	—	Komura et al. 2020
*T.cerebrata*	Brazil	INPA259601	KT287089	—	Komura et al. 2020
*T.crassicystidiata*	Brazil	INPA259607	KT287091	—	Komura et al. 2020
*T.crassicystidiata*	Brazil	INPA259606	KT287092	—	Komura et al. 2020
*T.griseibrunnea*	Brazil	INPA259610	KT287098	—	Komura et al. 2020
*T.griseibrunnea*	Brazil	INPA259608	KT287097	—	Komura et al. 2020
*T.griseibrunnea*	Brazil	INPA259609	KT287099	—	Komura et al. 2020
*T.nigripes*	Unknown	TOR89 (SFSU)	EF175540	—	Honan et al. 2015
*T.nigripes*	USA	TENN-F-060065	DQ449941	—	Lickey et al. 2003
*T.nigripes*	USA	TENN-F-060781	DQ449942	—	Lickey et al. 2003
*T.nigripes*	USA	TENN-F-060065	KT270853		Honan et al. 2015
*T.nigripes*	USA	MCA6925	MG717370	MG717355	Koch et al. 2018
*T.nigripes*	Not indicated	Wong888 (SFSU)	EF175535	—	Honan et al. 2015
*Tetrapyrgosnovinigripes*	Brazil	INPA259605	KT287082	—	Komura et al. 2020
*T.novinigripes*	Brazil	INPA259603	KT287083	—	Komura et al. 2020
*T.parvispora*	Thailand	AHH66	EF175536	—	Honan et al. 2015
*T.parvispora*	Thailand	AHH122 (SFSU)	EF175551	—	Honan et al. 2015
*T.parvispora*	Thailand	AHH26 (SFSU)	EF175546	—	Honan et al. 2015
*T.parvispora*	Thailand	AHH27 (SFSU)	EF175547	—	Honan et al. 2015
*T.parvispora*	Not indicated	DED7603 (SFSU)	KT270855	—	Honan et al. 2015
*T.similinigripes*	Brazil	INPA259600	KT287084	—	Komura et al. 2020
*T.similinigripes*	Brazil	INPA265162	KT287085	—	Komura et al. 2020
*T.similinigripes*	Brazil	INPA265320	KT287086	—	Komura et al. 2020
*T.* sp.	Costa Rica	TENN-F-056741	OQ171250	OQ171250	Petersen and Hughes 2024
*T.* sp.	Brazil	INPA265272	KT287100	—	Komura et al. 2020
*T.* sp.	Costa Rica	REHalling7542 (SFSU)	EF175531	—	Honan et al. 2015
*T.* sp.	USA	TENN-F-068199	OQ171255	OQ171255	Petersen and Hughes 2024
*T.* sp.	USA	TENN-F-068191	OQ171254	OQ171254	Petersen and Hughes 2024
*T.* sp.	Puerto Rico	TJB7902 (SFSU)	EF175542	—	Honan et al. 2015
*T.* sp.	Brazil	DLK1970	KT287101	—	Komura et al. 2020
*T.* sp.	Costa Rica	ZT12386 (SFSU)	EF175543		Honan et al. 2015
*T.* sp.	Australia	TENN-F-053779	OQ171239	—	Petersen and Hughes 2024
*T.* sp.	Costa Rica	TENN-F-056390	OQ171246	—	Petersen and Hughes 2024
*T.subcinerea*	Malaysia	KUM60047	EF175549	—	Honan et al. 2015
*T.subcinerea*	Malaysia	AHH84 (SFSU)	EF175524	—	Honan et al. 2015
*T.subcinerea*	USA	DED 6178 (SFSU)	EF175528	—	Honan et al. 2015
*T.subcinerea*	Thailand	AHH71 (SFSU)	EF175534	—	Honan et al. 2015
*T.subcinerea*	Indonesia	AR 019 (SFSU)	EF175548	—	Honan et al. 2015
*T.subcinerea*	Malaysia	KUM60047 (SFSU)	EF175549	—	Honan et al. 2015
*T.subcinerea*	Thailand	DED 7448 (SFSU)	EF175553	—	Honan et al. 2015
*T.subcinerea*	Indonesia	AR 505 (SFSU)	EF175530	—	Honan et al. 2015
*T.subcinerea*	Malaysia	DED 7517 (SFSU)	EF175532	—	Honan et al. 2015
*T.subcinerea*	Malaysia	AHH86 (SFSU)	EF175537	—	Honan et al. 2015
*T.subcinerea*	Indonesia	AHH115 (SFSU)	EF175550	—	Honan et al. 2015
*T.subcinerea*	Indonesia	AHH109 (SFSU)	EF175555	—	Honan et al. 2015
*T.subcinerea*	Malaysia	AHH90 (SFSU)	EF175552	—	Honan et al. 2015
*T.subcinerea*	Malaysia	RW832 (SFSU)	EF175539	—	Honan et al. 2015
*T.subcinerea*	Malaysia	KUM60051 (SFSU)	EF175527	—	Honan et al. 2015
*T.subcinerea*	Indonesia	AR 138 (SFSU)	EF175554	—	Honan et al. 2015
Uncultured Marasmiaceae clone	Unknown	OTU26	MH005865	—	Xing et al. 2019

## ﻿Results

### ﻿Molecular phylogeny

**Figure 1.**
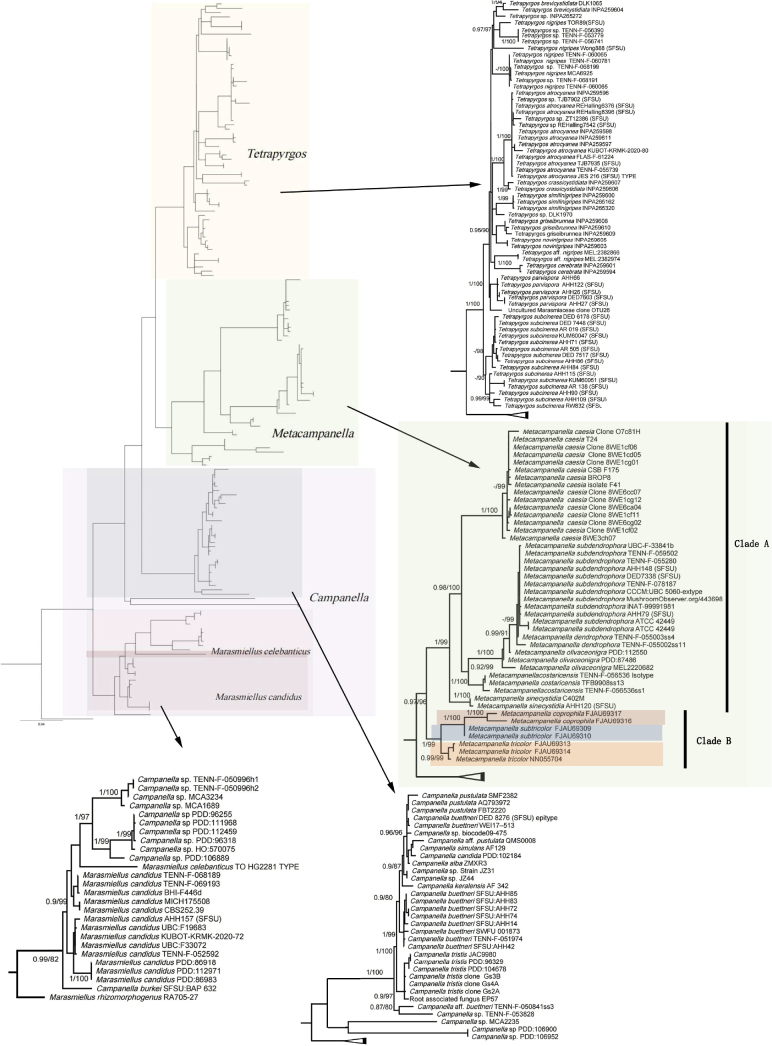
The phylogenetic relationships of *Tetrapyrgos*, *Campanella*, and *Metacampanella* based on nrITS plus LSU sequences. Nodes with PP (posterior probabilities) values ≥0.9 and ML bootstrap support values ≥80% are indicated in the phylogenetic tree. Sequences newly generated in this study are highlighted in different colored fonts. This ML tree generated by RAxML v8.2.4.

The results of molecular phylogenetic analysis showed that ML and BI have the same topological structure. The ML tree was shown in Fig. 1, and the self-reported values (MLbs ≥ 80%) and posterior probability values (PP ≥ 0.8) of ML tree and BI tree were marked on each branch. This phylogenetic tree was basically consistent with the tree of previous studies by J.J.S. Oliveira (Oliveira et al. 2020), Vladimír Antonín (Antonín et al. 2023) and Ronald H. Petersen (Petersen and Hughes 2024). The proposed new species *Metacampanellasubtricolor*T. Bau & W.N. Hou (PP = 1, MLbs = 100%) and *Metacampanellacoprophila* Burenbaatar Ganbaatar & T. Bau (PP = 1, MLbs = 100%) were both independent branches with high support and were sister groups to each other. A related taxon, the so-called *Marasmiellustricolor* (Alb. & Schwein.) Singer clustered in *Metacampanella* with high support (PP = 1, MLbs = 99%), so it should be treated as a member of *Metacampanella*.

### ﻿Taxonomy

#### ﻿ Metacampanella coprophila

Taxon classificationFungiAgaricalesMarasmiaceae

Burenbaatar Ganbaatar & T. Bau sp. nov.

0D561317-D711-5FAD-874F-00D9A089C913

 854569

Figs 2A, B , 3

##### Type material.

***Holotype*.** Mongolia • Dornod, Menengiin tal, 8 August 2023, 47°40'28"N, 116°50'21"E, alt. 600 m, Tolgor Bau & Burenbaatar Ganbaatar, BH34(FJAU69317).

**Figure 2.**
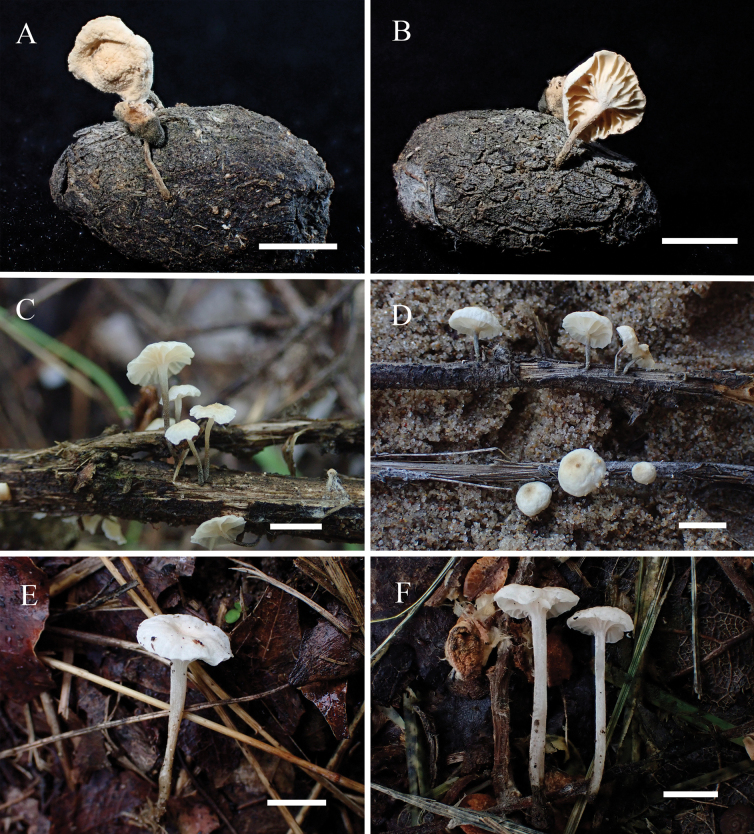
Basidiomata **A, B***Metacampanellacoprophila***C, D***Metacampanellasubtricolor***E, F***Metacampanellatricolor*. Scale bars: 5 mm.

##### Etymology.

“*coprophila*” refers to the growth on dung.

**Figure 3.**
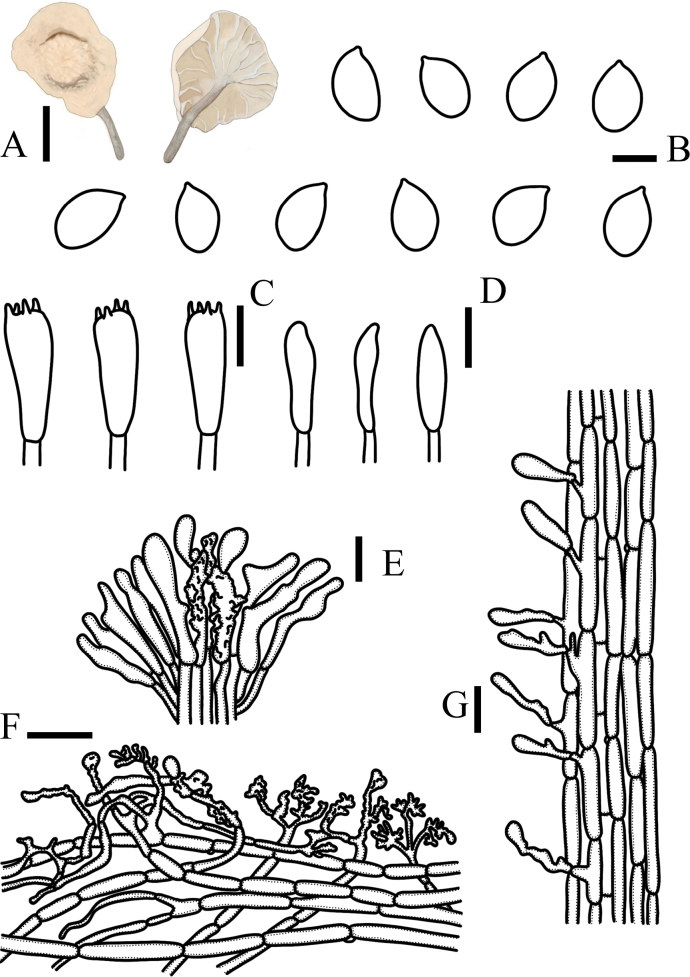
*Metacampanellacoprophila***A** basidiomata **B** basidiospores **C** basidia **D** basidioles **E** cheilocystidia **F** pileipellis **G** stipitipellis. Scale bars: 1 mm (**A**); 5 µm (**B**); 15 µm (**C–G**).

##### Diagnosis.

The pileus is white. The surface is frosted. The stipe is finely scaly, and the base is dark yellowish brown. Basidiospores are ellipsoid. Cheilocystidia two types: tetrapyrgoid, mostly ten pin-shaped. Caulocystidia is long clavate, irregularly curved, smooth.

##### Description.

Pileus 2–4 mm, bell-shaped when young, then applanate, central protuberance, the surface uneven like a grid pattern after drying, edge slightly involuted, membranous. The surface of the cap is milky white, creamy or beige, rough, frosted scales, the edge complete, wavy, without stripes. The flesh is white and thin. Lamellae white to creamy, distant (L = 12–14, I = 1–2), decurrent. Stipe 4–5 × 0.5–1 mm, cylindrical, upper and lower equal thick, hollow, fibrous, milky white at the top, yellowish white to yellowish brown in the middle, dark yellowish brown, brown to dark brown at the base, and white powdery frosty fine scales on the surface.

Basidiospores 7.8–10.5 × 5–7 μm, Q = 1.5, ellipsoid, the front non-protruding, colorless, smooth, transparent, thin-walled, non-amyloid. Basidia 26–37 × 6–8 μm, clavate, 4-spored, sometimes 2-spored, thin-walled, colorless, transparent, clamp connections present at base. Basidioles 25–37 × 4–6.8 μm, rod-shaped or spindle-shaped, thin-walled. Trama hyphae of lamellae irregularity, 3.2–4.6 μm in width, with clamp connections, thick-walled. Cheilocystidia two types: a. tetrapyrgoid, colorless, transparent, thick-walled, 16–21 × 5.7–6.3 µm, clavate or fusiform, an axis beset with numerous diverticula, a swollen obovate apex, smooth, rare; b. mostly ten pin-shaped, phialide or clavate, apex bifurcated or mid-divergent, smooth. Pleurocystidia absent. Terminal cellular elements of pileipellis two types: a. diverticulate repent hypha, 21–30 × 4.7–6.3 µm, light yellow, thick-walled. b. minority tetrapyrgoid, 20–27 × 6.7–7.4 µm, thick-walled. Pileipellis composed of extremely tightly interwoven hyphae, 4–6.9 µm in width, pale yellow, transparent, thick-walled. Caulocystidia 19–30 × 4.8–8 µm, phialide or clavate, irregularly curved, yellow, thick-walled, smooth. Stipitipellis cylindrical, 4.2–5.9 µm in width, parallel, yellow, thick-walled. Trama hyphae of stipe cylindrical, 6–8 µm in width, parallel, colorless, transparent, thick-walled. Clamp connections present in all tissues.

##### Habitat and distribution.

Summer-growing, in sheep dung. Currently only known from Mongolia.

##### Additional specimens examined.

Mongolia • Dornod, Menengiin tal, 8 August 2023, 47°40'28"N, 116°50'20"E, Tolgor Bau, Haiying Bao and Burenbaatar Ganbaatar, B34(FJAU69316).

##### Notes.

This species is similar to *Metacampanellasubtricolor* in terms of morphological characteristics. However, cheilocystidia of *Metacampanellacoprophila* is two types: a. tetrapyrgoid, b. mostly ten pin-shaped and caulocystidia only phialide or clavate. Nevertheless cheilocystidia of *Metacampanellasubtricolor* is only tetrapyrgoid and caulocystidia two types: a. tetrapyrgoid, b. individual arboreal dermatocystidia. At the same time, the habitat of *Metacampanellacoprophila* is special for sheep dung.

#### ﻿ Metacampanella subtricolor

Taxon classificationFungiAgaricalesMarasmiaceae

T. Bau & W. N. Hou sp. nov.

3D2A41CF-7FF0-58A9-A1BC-9B6C4E30DD0E

 854571

Figs 2C, D , 4

##### Type material.

***Holotype*.** China • Inner Mongolia, Tongliao City, Daqinggou national nature reserve, 16 July 2023, 42°49′20″N, 122°15′42″E, alt. 313 m, Weinan Hou, H2307120(FJAU69309)

**Figure 4.**
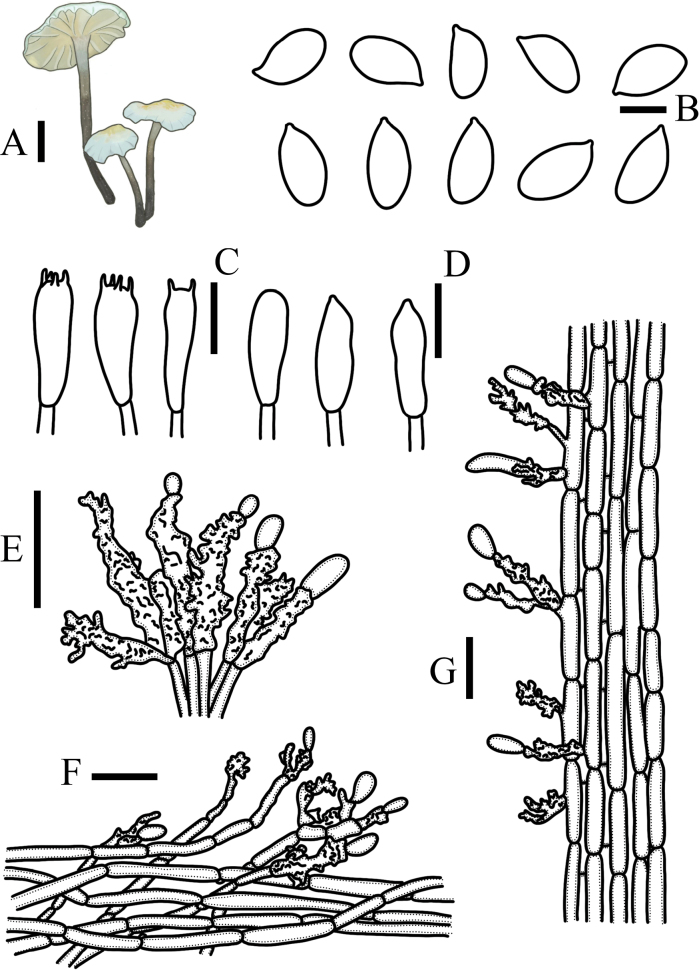
*Metacampanellasubtricolor***A** basidiomata **B** basidiospores **C** basidia **D** basidioles **E** cheilocystidia **F** pileipellis **G** stipitipellis. Scale bars: 1 mm (**A**); 5 µm (**B**); 15 µm (**C–G**).

##### Etymology.

“*subtricolor*” refers that this species is similar to *Metacampanellatricolor*.

##### Diagnosis.

Pileus is milky white, with furrowed edges and frosted surface. The stipe is finely scaly and black at base. Basidiospores are ellipsoid. Cheilocystidia tetrapyrgoid. Caulocystidia two types: a. tetrapyrgoid, b. individual arboreal dermatocystidia.

##### Description.

Pileus 3–6 mm, bell-shaped to hemispherical when young, and then gradually flattened, with a slight uplift in the center and wavy edges. The surface of the cap is densely covered with white powder frost, membranous, milky white to light yellow when young, light yellowish brown to brown in the center when mature, white to light yellow on the edge, with shallow grooves, membranous. Context milky white, thin. Lamellae cream to light yellowish brown, distant (L = 10–12, I = 1–2), decurrent. Stipe 4–6 × 1–2 mm thick, cylindrical, equal thick or tapering downward. The top of the stipe milky white to light yellow brown, the middle is brown, slightly transparent, and the base dark brown to black and the surface white fine scales, fibrous, hollow.

Basidiospores 7.7–10 × 4.5–6 µm, Q = 1.7, ellipsoid, colorless, transparent, thin-walled, non-amyloid. Basidia 24–30 × 8–11 µm, clavate, 4(2)-spored, colorless, transparent, thin-walled, base with clamp connections. Basidioles 20–23 × 5–8 µm, clavate or subfusiform, thin-walled. Trama hyphae of lamellae irregularity, 3–4.9 µm in width, with clamp connections and thick-walled. Cheilocystidia tetrapyrgoid, 18–27 × 5–8 µm, cylindrical, apex with or without a swollen obovate, base irregularly branched, surface protrusions, colorless, transparent, thick-walled. Pleurocystidia absent. Terminal cellular elements of pileipellis tetrapyrgoid, 20–36 × 4–7 µm, colorless, thick-walled. Pileipellis interwoven hyphae, 3.4–5.6 µm in width, pale yellow, transparent, verrucous surface, thick-walled. Caulocystidia two types: a. tetrapyrgoid, 11–22 × 4–7 µm, pale yellow, thick-walled, cylindrical, with spherical or ellipsoid cells at the top and protrusions on the surface; b. individual arboreal dermatocystidia. Stipitipellis composed of cylindrical hyphae, 2.9–4.7 µm, parallel, yellowish brown, thick-walled. Trama hyphae of stipe composed of cylindrical hyphae, 6.2–8 µm in width, parallel, colorless, transparent, thick-walled. Clamp connections present in all tissues.

##### Habitat and distribution.

Summer-growing, on the residues of *Artemisiahalodendron* Turcz.ex Bess. Currently only known from northeast China.

##### Additional specimens examined.

**China** • Inner Mongolia, Tongliao City, Naiman Banner, Xinglongnuma Forest Farm, 12 July 2022; 43°23′31″N, 122°12′20″E, alt. 329 m, Tolgor Bau, Fang Guo, gf2234 (FJAU67070). China • Inner Mongolia, Tongliao City, horqin left back banner, Nugustai Town, 14 July 2022, 43°23′48″N, 122°24′53″E, alt. 311 m, Tolgor Bau, Weinan Hou, H220752 (FJAU67068). China • Inner Mongolia, Daqinggou national nature reserve, 16 July 2023, 42°49′18″N, 122°15′31″E, alt. 312 m, Tolgor Bau, Weinan Hou, H2411102 (FJAU69310).

##### Notes.

This species is similar to *Metacampanellasubtricolor* with respect to morphological characteristics. Caulocystidia of *Metacampanellasubtricolor* comprise two types: a. tetrapyrgoid, b. individual arboreal dermatocystidia, but caulocystidia of *Metacampanellatricolor* is composed of three types: a. long cylindrical or long clavate, b. U-shape, c. a few short clavate, base with irregular protrusion.

#### ﻿ Metacampanella tricolor

Taxon classificationFungiAgaricalesMarasmiaceae

(Alb. & Schwein.) T. Bau & W. N. Hou comb. nov.

1872075C-7C15-527F-901D-8F3AAD0EFC22

 854570

Figs 2E, F , 5

Agaricus tricolor Alb. & Schwein., Consp. fung. (Leipzig): 224 (1805) Basionym.
Marasmiellus tricolor (Alb. & Schwein.) Singer, Pap. Mich. Acad. Sci. 32: 128 (1948)[1946] Synonym.

##### Type material.

***Holotype*.** China • Inner Mongolia, Tongliao City, horqin left back banner, Udantara Forest Farm, 15 July 2023, 43°1′17″N, 122°44′47″E, alt. 345 m, Weinan Hou, H230754(FJAU69314).

**Figure 5.**
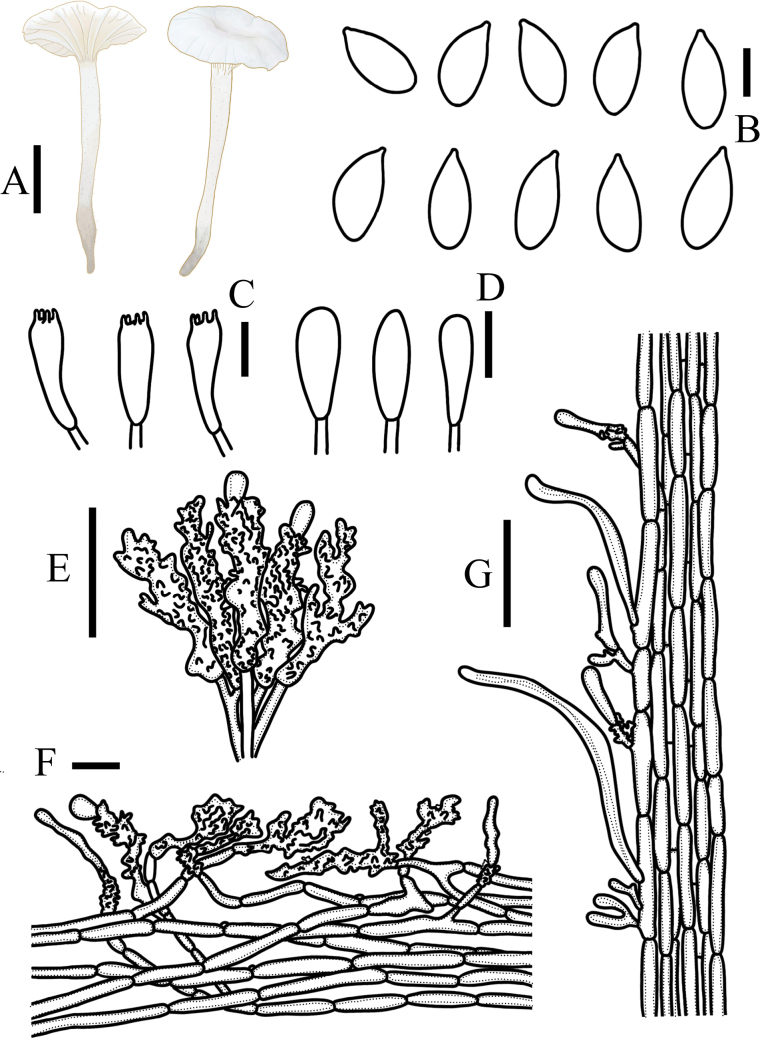
*Metacampanellatricolor***A** basidiomata **B** basidiospores **C** basidia **D** basidioles **E** cheilocystidia **F** pileipellis **G** stipitipellis. Scale bars: 2 mm (**A**); 5 µm (**B**); 15 µm (**C–G**).

##### Description.

Pileus 4–10 mm, convex when young, applanate after maturity, slightly concave at the center, and shallow grooves on the edge. The surface of the pileus white, creamy, center light yellow, with pink tone, rough, white powder frosty fine scale, membranous. Context white, thin. Lamellae rice white to cream color, distant(L = 8–10, I = 1–2), decurrent. Stipe 6–24 × 1–2 mm, cylindrical, slightly thinner at the base, white at the top of the stipe, light yellow in the middle, brown to grayish brown at the base, densely white fine scale on the surface, fibrous, hollow.

Basidiospores 8.6–11.8 × 4.8–6.3 µm, Q = 1.9, long ellipsoid, not protuberant on the front, slightly sharp at base, colorless, transparent, thin-walled, non-amyloid. Basidia 22–32 × 7.4–11 µm, clavate, 4(2)-spored, colorless, transparent, thin-walled, base with clamp connections. Basidioles 20–30 × 5–10 µm, clavate or subfusiform, thin-walled. Trama hyphae of lamellae irregularity, 3.8–5.7 µm in width, with clamp connections, thick-walled. Cheilocystidia tetrapyrgoid, 18–27 × 5–8 µm, the main body cylindrical, irregular protrusion on the surface. Pleurocystidia absent. Terminal cellular elements of pileipellis two types: a. tetrapyrgoid, 30–47 × 6–12 µm; b. long cylindrical or long clavate, verrucous base, colorless, 37–49 × 4.4–6 µm, thick-walled. Caulocystidia three types: a. long cylindrical or long clavate, thick-walled, smooth, 23–59 × 3.7–6.6 µm; b. U-shaped, smooth, 16–28 × 3.3–6.7 µm; c. a few short clavate, base with irregular protrusion, 16–29 × 3–6.8 µm. Stipitipellis composed of cylindrical hyphae, 3.3–5.6 µm in width, parallel, pale yellow, thick-walled. Stipitipellis composed of cylindrical hyphae, 4–6 µm in width, parallel, colorless, transparent, thick-walled. Trama hyphae of stipe composed of cylindrical hyphae, 3.3–5.6 µm in width, parallel, transparent, colorless, thick-walled. Clamp connections present in all tissues.

##### Habitat and distribution.

Summer-growing, on the residues of herbaceous plants. Known from south, northwest and northeast China.

##### Additional specimens examined.

China • Inner Mongolia, Tongliao City, Liaohe Park, 19 July 2023, 43°39′10″N, 122°16′57″E, alt. 179 m, Tolgor Bau and Weinan Hou, H2307216(FJAU69318). China • Inner Mongolia, Tongliao City, horqin left back banner, Udantara Forest Farm, 15 July 2023, 43°1′17″N, 122°44′47″E, alt. 345 m, Weinan Hou, H230759(FJAU69313). China • Heilongjian, Yichun City, Beishan Park, 25 July 2023, 47°44′13″N, 128°53′18″E, alt. 318 m, Weinan Hou, H2307334(FJAU69315). China • Liaoning, Zhuanghe City, Xishan Park, 6 July 2024, 39°37′51″N, 121°59′51″E, alt. 70 m, Weinan Hou, H2470619(FJAU69319). China • Liaoning, Dalian City, Heping Park, 9 July 2024, 38°47′22″N, 121°8′57″E, Hong Cheng, C24070901(FJAU69320).

##### Notes.

Because the macroscopic morphological characteristics of *Metacampanellatricolor* are very close to species of *Marasmiellus*, Singer classified this species into *Marasmiellus* in the early stage (Singer 1948). However, now it was observed that the microstructure characteristics of pileipellis and cheilocystidia were more in line with the definition of *Metacampanella* (Petersen and Hughes 2024).

### ﻿Key to the species of *Metacampanella*

**Table:** 

1	hymenophore usually of some radial rounded veins, usually developing few to numerous interveins, meandering or joining the major veins	**2**
–	hymenophore lamellae, none radial rounded vein	**8**
2	sessile, pseudostipitate, laterally stipitate (<1 mm	**3**
–	well-developed stipe(≥1 mm	**6**
3	surface of pileus gelatinous	***Me.dendrophora***
–	surface of pileus non-gelatinous	**4**
4	basidiospores triangular or round triangular, with abaxial bulg-e	***Me.subdendrophora***
–	basidiospores ellipsoid, without abaxial bulge	**5**
5	cheilocystidia missing	***Me.caesia***
–	cheilocystidia ten pin-shaped, tetrapyrgoid or gymnopoi-d	**Me.dendrophoraf.washingtonensis**
6	surface of pileus uniform dark green, not white	***Me.costaricensis***
–	surface of pileus white, with suffused greenish	**7**
7	pileipellis hyphae Rameales-structure	***Me.sinecystidia***
–	pileipellis hyphae not Rameales-structure	***Me.olivaceonigra***
8	grow in sheep dung	***Me.coprophila***
–	grow on branches of trees or dead grass	**9**
9	Caulocystidia three types: a. long cylindrical or long clavate, b. U-shaped, c. a few short clavate, base with irregular protrusion	***Me.tricolor***
–	Caulocystidia two types: a. tetrapyrgoid b. individual arboreal dermatocystidi-a	***Me.subtricolor***

## ﻿Discussion

Peterson’s phylogenetic framework combined with sample sequences from China and Mongolia was used to reconstruct a phylogenetic tree based on the ITS and nrLSU datasets. The new species, i.e., *Metacampanellacoprophila*, identified in this study formed an independent evolutionary branch in the phylogenetic tree and a sister group with *Metacampanellasubtricolor*. However, there are some differences in their habitats. The former was isolated from sheep dung and the latter from herbaceous plant residues. *Metacampanellatricolor* was originally placed in *Marasmiellus* (Singer 1948). In the present study, morphological observations and phylogenetic analysis showed that this species belongs to the genus *Metacampanella*, with high phylogenetic support - (PP = 0.99, MLbs = 97%). It is a sister group of *Metacampanellasubtricolor* and *Metacampanellacoprophila*. This species was widely distributed in Asia, and also distributed in south, northwest and northeast China (Song et al. 2009; Luo 2021).

In the phylogenetic tree of this study, there were two major branches Clade A and Clade B. The main difference between these clades was that the hymenia of Clade A had some radial rounded veins, and the hymenia of Clade B had real lamellae. Our samples belonged to Clade B, which was located at the base of *Metacampanella*. Their stipe length was ≥ 1 mm, which was similar to *Me.costaricensis*, *Me.olivaceonigra*, and *Me.sinecystidia* in Clade A. Basidiospores were ellipsoid, with no protuberant on the front, similar to *Me.caesia* in Clade A. In addition, the recognition characteristics of basidiospore ellipsoid in this branch species were also similar to *Campanella*. The basidiophore was umbellate, the pileus was convex, not conchate or obcupulate-to-obsaucer in shape, and the hymenium was lamellar. These characteristics are similar to those of *Tetrapyrgos*. However, the basidiospores of *Tetrapyrgos* were quadrilateral, and the species in Clade B did not meet the definition of *Tetrapyrgos*. In addition, we speculate that the differentiation of the basidiospore morphology of these genera occurred earlier than that of other characteristics. Clade B reflects the different genetic characteristics of these genera and plays a vital role in the classification of these species. This provides key clues for determining the species boundary of *Metacampanella* and *Tetrapyrgos*, and the multilineage problem of *Campanella*.

The original identifying characteristics of *Metacampanella* have changed, because Clade B has broadened and increased the species range of *Metacampanella*. We attempted to supplement and revise the definition of species characteristics in *Metacampanella*. After revision, the characteristics of the genus were basidiophore conchate or obcupulate to obsaucer-shaped or umbellate, white to suffused greenish or yellowish (rarely pale pink), membrane, sometimes gelatinous, hymenium some radial rounded veins or lamellae, sessile, pseudostipitate, laterally stipitate or well-developed stipe, finely scales on the surface, necropigment at the base, basidiospores generally triangular, round triangular or ellipsoid, sometimes with or without abaxial bulge, cheilocystidia missing, ten pin-shaped, tetrapyrgoid, gymnopoid or metuloid, with or without crystalline deposit, pileipellis a thatch of slender hyphae, often encrusted with annular thickenings, pileocystidia tetrapyrgoid, long cylindric or missing, caulocystidia long cylindrical or long clavate, U-shaped, short clavate with irregular protrusion at base, tetrapyrgoid, individual arboreal dermatocystidia. Growing on dead woody substrates, dead grass, a few on dung.

This study provides specimens and their corresponding sequences from three species of *Metacampanella* from China and Mongolia. Because we only referred to the results of the species at the time of establishment of the genus, we did not modify the level of the subgenus. We have only provided key information and compiled a key for the species of this genus.

## Supplementary Material

XML Treatment for Metacampanella coprophila

XML Treatment for Metacampanella subtricolor

XML Treatment for Metacampanella tricolor

## ﻿Additional information

### Author ORCIDs

Wei-nan Hou https://orcid.org/0000-0002-9838-3489

Burenbaatar Ganbaatar https://orcid.org/0000-0003-0720-2441

Tolgor Bau https://orcid.org/0000-0003-2461-9345

### Data availability

All of the data that support the findings of this study are available in the main text.

## References

[B1] Aime MC Phillips-Mora W (2005) The causal agents of witches’ broom and frosty pod rot of cacao (chocolate, Theobroma cacao) form a new lineage of Marasmiaceae. Mycologia 97(5): 1012–1022. 10.3852/mycologia.97.5.1012 16596953

[B2] Alanbagi RA Alshuwaili FE Stephenson SL (2019) Fungi associated with forest floor litter in northwest Arkansas. Current Research in Environmental & Applied Mycology 9(1): 25–35. 10.5943/cream/9/1/3

[B3] Alzohairy AM (2011) BioEdit: An important software for molecular biology. GERF Bulletin of Biosciences 2(1): 60–61.

[B4] Antonín V Hosaka K Kolařík M (2023) Taxonomy and phylogeny of *Paramarasmius* gen. nov. and *Paramarasmiusmesosporus*, a world wide distributed fungus with a strict ecological niche. Plant Biosystems - An International Journal Dealing with all Aspects of Plant Biology 157(8): 1–12. 10.1080/11263504.2022.2100503

[B5] Bau T Liu Y (2010) *Campanella* in China. Journal of Fungal Research 8(1): 19–22. 10.1080/10670560903335728

[B6] Bougoure DS Cairney JW (2005) Assemblages of ericoid mycorrhizal and other root-associated fungi from *Epacrispulchella* (Ericaceae) as determined by culturing and direct DNA extraction from roots. Environmental Microbiology 7(6): 819–827. 10.1111/j.1462-2920.2005.00755.x 15892701

[B7] Cai Q Cui YY Yang ZL (2016) Lethal *Amanita* species in China. Mycologia 108(5): 993–1009. 10.3852/16-008 27474516

[B8] Cui YY Cai Q Tang L Liu JW Yang ZL (2018) The family Amanitaceae: Molecular phylogeny, higher-rank taxonomy and the species in China. Fungal Diversity 91(1): 5–230. 10.1007/s13225-018-0405-9

[B9] David AS Seabloom EW May G (2016) Plant host species and geographic distance affect the structure of aboveground fungal symbiont communities, and environmental filtering affects belowground communities in a coastal dune ecosystem. Microbial Ecology 71(4): 912–926. 10.1007/s00248-015-0712-6 26626912

[B10] Dearnaley JDW Bougoure JJ (2010) Isotopic and molecular evidence for saprotrophic Marasmiaceae mycobionts in rhizomes of *Gastrodiasesamoides*. Fungal Ecology 3(4): 288–294. 10.1016/j.funeco.2009.11.003

[B11] Desjardin DE Perry BA Shay JE Newman DS (2017) The type species of *Tetrapyrgos* and *Campanella* (Basidiomycota, Agaricales) are redescribed and epitypified. Mycosphere: Journal of Fungal Biology 8(8): 977–985. 10.5943/mycosphere/8/8/1

[B12] Doyle JJ Doyle JL (1987) A rapid DNA isolation procedure for small quantities of fresh leaf tissue. Phytochemical Bulletin 19: 11–15.

[B13] Guan QX Zhao CL (2021) Taxonomy and phylogeny of the wood-inhabiting fungal genus *Hyphoderma* with descriptions of three new species from East Asia. Journal of Fungi (Basel, Switzerland) 7(4): 308. 10.3390/jof7040308 33923807 PMC8072537

[B14] Haelewaters D Dirks AC Kappler LA Mitchell JK Quijada L Vandegrift R Buyck B Pfister DH (2018) A preliminary checklist of fungi at the Boston harbor islands. Northeastern Naturalist 9(Special Issue): 45. 10.1656/045.025.s904

[B15] Hennings PC (1895) Fungi camerunenses I. Botanische Jahrbücher für Systematik, Pflanzengeschichte und Pflanzengeographie 22: 72–111.

[B16] Herrera J Khidir HH Eudy DM Porras-Alfaro A Natvig DO Sinsabaugh RL (2010) Shifting fungal endophyte communities colonize *Boutelouagracilis*: Effect of host tissue and geographical distribution. Mycologia 102(5): 1012–1026. 10.3852/09-264 20943502

[B17] Herrera J Poudel R Khidir HH (2011) Molecular characterization of coprophilous fungal communities reveals sequences related to root-associated fungal endophytes. Microbial Ecology 61(2): 239–244. 10.1007/s00248-010-9744-0 20842497

[B18] Honan AH Desjardin DE Perry BA Baroni HE Baroni TJ (2015) Towards a better understanding of *Tetrapyrgos* (Basidiomycota, agaricales): New spe-cies, type studies, and phylogenetic inferences. Phytotaxa 231(2): 101–132. 10.11646/phytotaxa.231.2.1

[B19] Horak E (1986) *Tetrapyrgos* Horak (nom. et gen. nov.) replacing Pterospora Métrod (1949; nom. preocc.). Sydowia 39: 101–103. 10.3389/fmicb.2018.03321

[B20] Kalyaanamoorthy S Minh BQ Wong TKF von Haeseler A Jermiin LS (2017) ModelFinder: Fast model selection for accurate phylogenetic estimates. Nature Methods 14(6): 587–589. 10.1038/nmeth.4285 28481363 PMC5453245

[B21] Kantharaja R Krishnappa M (2020) Morphological and molecular phylogenetic studies on *Battarreaphalloides* (Agaricales): A new report to Indian mycobiota. Journal of Threatened Taxa 12(8): 15881. 10.11609/jott.5679.12.8.15881-15888

[B22] Kantharaja R Krishnappa M (2022) Amanitaceous fungi of central Western Ghats: Taxo­nomy, phylogeny, and six new reports to Indian mycobiota. Journal of Threatened Taxa 14(4): 20890–20902. 10.11609/jott.7801.14.4.20890-20902

[B23] Kirk PM Cannon PF Minter DW Stalpers JA (2008) Ainsworth & Bisby′s dictionary of the fungi, 10^th^ ed. Wallingford, CABI. 10.1079/9780851998268.0000

[B24] Koch RA Lodge DJ Sourell S Nakasone K McCoy AG Aime MC (2018) Tying up loose threads: Revised taxonomy and phylogeny of an avian-dispersed Neotropical rhizomorph-forming fungus. Mycological Progress 17(9): 989–998. 10.1007/s11557-018-1411-8

[B25] Komura DL Oliveira JJS Moncalvo J Margaritesco S Zartman C (2020) Six new species of *Tetrapyrgos* (Basidiomycota, Agaricales, Marasmiaceae) from the Brazilian Amazon. Phytotaxa 440: 193–214. 10.11646/phytotaxa.440.3.2

[B26] Kornerup A Wanscher JH (1978) Methuen Handbook of Colour, 3^rd^ ed. Methuen, London, UK, 252 pp.

[B27] Larsson E Örstadius L (2008) Fourteen coprophilous species of Psathyrella ide-ntified in the Nordic countries using morphology and nuclear rDNA seque-nce data. Mycological Research 112(10): 1165–1185. 10.1016/j.mycres.2008.04.003 18707856

[B28] Lickey EB Hughes KW Petersen RH (2003) Phylogenetic and taxonomic studies in Artomyces and Clavicorona (Homobasidiomycetes: Auriscalpiaceae). Sydowia 55(2): 181–254.

[B29] Luo Y (2021) Studies on Macrofungal Diversity and Mycomedicine Resources in Jiaohe Region, Jilin Province. Jilin Agricultural University.

[B30] Mou GF Bau T (2021) Asproinocybaceae fam. nov. (Agaricales, Agaricomycetes) for Accommodating the Genera *Asproinocybe* and *Tricholosporum*, and Description of *Asproinocybesinensis* and *Tricholosporumguangxiense* sp. nov. Journal of Fungi (Basel, Switzerland) 7(12): 1086. 10.3390/jof7121086 34947067 PMC8707192

[B31] Oliveira JJS Moncalvo JM Margaritescu S Capelari M (2020) A morphological and phylogenetic evaluation of Marasmiussect.Globulares (*Globulares*-*Sicci* complex) with nine new taxa from the Neotropical Atlantic Forest. Persoonia 44(1): 240–277. 10.3767/persoonia.2020.44.09 33116342 PMC7567966

[B32] Osmundson TW Bergemann SE Rasmussen R Garbelotto MM (2022) Using point data to assess biogeographical signal, endemicity and factors associated with macrofungal diversity in the data‐poor Pacific oceanic island bioregion. Journal of Biogeography 49(5): 1–13. 10.1111/jbi.14354

[B33] Pereira E Vázquez de Aldana BR San Emeterio L Zabalgogeazcoa I (2019) A Survey of culturable fungal endophytes from Festucarubrasubsp.pruinosa, a grass from marine cliffs, reveals a core microbiome. Frontiers in Microbiology 9: 3321. 10.3389/fmicb.2018.03321 PMC6343541 30700985

[B34] Perez-de Gregorio MA Vizzini A Contu M Roque C Ercole E (2011) *Marasmielluscelebanticus* (Agaricales, Omphalotaceae), a new species of Marasmiellussect.Candidi collected in the Mediterranean area. Phytotaxa 25: 49–59. https://phytotaxa.mapress.com/pt/article/view/phytotaxa.25.1.6

[B35] Petersen RH Hughes KW (2024) *Metacampanella* gen. nov.: The *Campanelladendrophora* complex. Mycology: 1–28. 10.1080/21501203.2024.2309898 PMC11899269 40083406

[B36] Schoch CL Seifert KA Huhndorf S Robert V Spouge JL Levesque CA Chen W Bolchacova E Voigt K Crous PW Miller AN Wingfield MJ Aime MC An K-D Bai F-Y Barreto RW Begerow D Bergeron M-J Blackwell M Boekhout T Bogale M Boonyuen N Burgaz AR Buyck B Cai L Cai Q Cardinali G Chaverri P Coppins BJ Crespo A Cubas P Cummings C Damm U de Beer ZW de Hoog GS Del-Prado R Dentinger B Diéguez-Uribeondo J Divakar PK Douglas B Dueñas M Duong TA Eberhardt U Edwards JE Elshahed MS Fliegerova K Furtado M García MA Ge Z-W Griffith GW Griffiths K Groenewald JZ Groenewald M Grube M Gryzenhout M Guo L-D Hagen F Hambleton S Hamelin RC Hansen K Harrold P Heller G Herrera C Hirayama K Hirooka Y Ho H-M Hoffmann K Hofstetter V Högnabba F Hollingsworth PM Hong S-B Hosaka K Houbraken J Hughes K Huhtinen S Hyde KD James T Johnson EM Johnson JE Johnston PR Jones EBG Kelly LJ Kirk PM Knapp DG Kõljalg U Kovács GM Kurtzman CP Landvik S Leavitt SD Liggenstoffer AS Liimatainen K Lombard L Luangsa-ard JJ Lumbsch HT Maganti H Maharachchikumbura SSN Martin MP May TW McTaggart AR Methven AS Meyer W Moncalvo J-M Mongkolsamrit S Nagy LG Nilsson RH Niskanen T Nyilasi I Okada G Okane I Olariaga I Otte J Papp T Park D Petkovits T Pino-Bodas R Quaedvlieg W Raja HA Redecker D Rintoul TL Ruibal C Sarmiento-Ramírez JM Schmitt I Schüßler A Shearer C Sotome K Stefani FOP Stenroos S Stielow B Stockinger H Suetrong S Suh S-O Sung G-H Suzuki M Tanaka K Tedersoo L Telleria MT Tretter E Untereiner WA Urbina H Vágvölgyi C Vialle A Vu TD Walther G Wang Q-M Wang Y Weir BS Weiß M White MM Xu J Yahr R Yang ZL Yurkov A Zamora J-C Zhang N Zhuang W-Y Schindel D (2012) Nuclear ribosomal internal transcribed spacer (ITS) region as a universal DNA barcode marker for fungi. Proceedings of the National Academy of Sciences of the United States of America 109(16): 6241–6246. 10.1073/pnas.1117018109 22454494 PMC3341068

[B37] Singer R (1948) New and interesting species of Basidiomycetes. II. Papers of the Michigan Academy of Sciences 32: 103–150.

[B38] Singer R (1975) The neotropical species of *Campanella* and *Aphyllotus* with notes on some species of *Marasmiellus*. Nova Hedwigia 26L: 847–895.

[B39] Singer R (1986) The Agaricales in Modern Taxonomy. [M]. Koeltz Scientific Books, 981 pp.

[B40] Song B Deng CY Wu XL Li TH (2009) Known species of *Marasmius* from China and the their distribution. Guizhou Science 27(1): 1–18.

[B41] Vilgalys R Hester M (1990) Rapid genetic identification and mapping of enzymatically amplified ribosomal DNA from several *Cryptococcus* species. Journal of Bacteriology 172(8): 4239–4246. 10.1128/jb.172.8.4238-4246.1990 PMC213247 2376561

[B42] Vinnere O Fatehi J Sivasithamparam K Gerhardson B (2005) A new plant pathogenic sterile white basidiomycete from Australia. European Journal of Plant Pathology 112(1): 63–77. 10.1007/s10658-005-2191-y

[B43] Vu D Groenewald M Vries MD Gehrmann T Stielow B Eberhardt U Al-Hatmi A Groenewald JZ Cardinali G Houbraken J Boekhout T Crous PW Robert V Verkley GJM (2019) Large-scale generation and analysis of filamentous fungal DNA barcodes boosts coverage for kingdom fungi and reveals thresholds for fungal species and higher taxon delimitation. Studies in Mycology 92(1): 135–154. 10.1016/j.simyco.2018.05.001 29955203 PMC6020082

[B44] Wei CL Ke SY Wu SH (2021) Seven species of lignicolous agarics (Agaricomycetidae) new to Taiwan. Fungal Science 36(1): 15–22.

[B45] White TJ Bruns T Lee S Taylor JW (1990) Amplification and direct sequencing of fungal ribosomal RNA genes for phylogenetics. In: Innis MA Gelfand DH Sninsky JJ White TJ (Eds) PCR protocols: a guide to methods and applications.Academic Press, New York, 315–322. 10.1016/B978-0-12-372180-8.50042-1

[B46] Xing X Gai X Jacquemyn H Chen Y Gao Y Liu Q Guo S (2019) The impact of life form on the architecture of orchid mycorrhizal networks in tropical forest. Oikos 128(9): 1254–1264. 10.1111/oik.06363

[B47] Zhang D Gao F Jakovlić I Zou H Zhang J Li WX Wang GT (2020) PhyloSuite: An integrated and scalable desktop platform for streamlined molecular sequence data management and evolutionary phylogenetics studies. Molecular Ecology Resources 20(1): 348–355. 10.1111/1755-0998.13096 31599058

